# Sustained-release study on Exenatide loaded into mesoporous silica nanoparticles: in vitro characterization and in vivo evaluation

**DOI:** 10.1186/s40199-017-0186-9

**Published:** 2017-09-04

**Authors:** Cuiwei Chen, Hongyue Zheng, Junjun Xu, Xiaowei Shi, Fanzhu Li, Xuanshen Wang

**Affiliations:** 1 Department of Pharmaceutics, Zhejiang Chinese Medical University, Hangzhou, 311042 China; 2 Libraries of Zhejiang Chinese Medical University, Zhejiang Chinese Medical University, Hangzhou, 310053 China; 3 Department of Pharmacy, The Second Affiliated Hospital, School of Medicine, Zhejiang University, Hangzhou, 310052 China; 4 Department of Pharmacy, The Second Hospital of Dalian Medical University, Dalian, 116027 China

**Keywords:** Exenatide, Type 2 diabetic, Mesoporous silica nanoparticles, Sustained release, Pharmacokinetics/Pharmacodynamics

## Abstract

**Background:**

Exenatide (EXT), the first glucagon-like peptide-1 receptor agonist, has been approved as an adjunctive therapy for patients with type 2 diabetes. Due to EXT’s short half-life, EXT must be administrated by continuous subcutaneous (s.c.) injection twice daily. In previous studies, many studies on EXT loaded into polymer materials carriers for sustained release had been reported. However, these carriers have some defects, such as hydrophobicity, low surface energy, low mechanical strength, and poor chemical stability. Therefore, this study aims to develop a novel drug delivery system, which is EXT loaded into well-ordered hexagonal mesoporous silica structures (EXT-SBA-15), to control the sustainability of EXT.

**Methods:**

SBA-15 was prepared by hydrothermal method with uniform size. Morphology of SBA-15 was employed by transmission electron microscopy. The pore size of SBA-15 was characterized by N_2_ adsorption–desorption isotherms. The in vitro drug release behavior and pharmacokinetics of EXT-SBA-15 were investigated. Furthermore, the blood glucose levels of diabetic mice were monitored after subcutaneous injection of EXT-Sol and EXT-SBA-15 to evaluate further the stable hypoglycemic effect of EXT-SBA-15.

**Results:**

EXT-SBA-15 showed a higher drug loading efficiency (15.2 ± 2.0%) and sustained-release features in vitro. In addition, pharmacokinetic studies revealed that the EXT-SBA-15 treatment group extended the half-life *t*_1/2(β)_ to 14.53 ± 0.70 h compared with that of the EXT solution (EXT-Sol) treatment group (0.60 ± 0.08 h) in vivo. Results of the pharmacodynamics study show that the EXT-SBA-15 treatment group had inhibited blood glucose levels below 20 mmol/L for 25 days, and the lowest blood glucose level was 13 mmol/L on the 10th day.

**Conclusions:**

This study demonstrates that the EXT-SBA-15 delivery system can control the sustainability of EXT and contribute to improve EXT clinical use.

**Graphical abstract:**

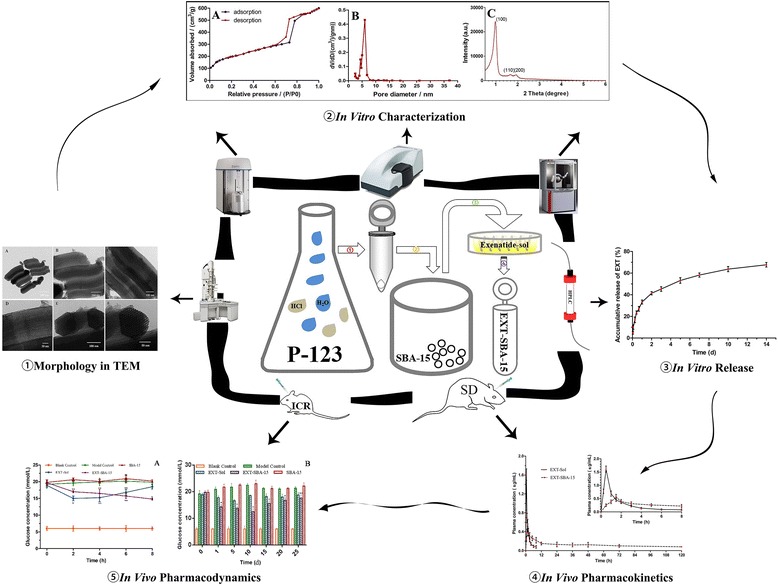

## Background

Exenatide (EXT), a 39-amino acid peptide, is the synthetic version of exendin-4 isolated from salivary secretions of *Heloderma horridum lizard* venom [1, 2]. EXT shares approximately 53% sequence homology with the mammalian glucagon-like peptide-1 (GLP-1), which exhibits obvious advantages developed as a glucose-lowering agent with similar functions as GLP-1 [3, 4]. In currently, EXT is available in the market (Byetta™) as adjunctive therapy to improve glucose homeostasis in type 2 diabetic patients [5]. However, the twice daily subcutaneous (s.c.) injections of EXT have posed clear shortcomings, such as inconvenience for the administration, local pain and irritation during the injection [6]. Sustained-release drug delivery system for exenatide (EXT) must be developed to solve the problem of long-term medication and overcome the inconvenience of injection.

Many studies had reported EXT loaded into carriers for sustained release [7–9]. The US FDA has approved a long-acting formulation of EXT dispersed in poly-(d,l-lactide-co-glycolide) polymer microspheres in 2012 [10]. However, these polymer-material carriers have defects, such as hydrophobicity, low surface energy, low mechanical strength, and poor chemical stability. In this study, we selected well-ordered hexagonal mesoporous silica structures (SBA-15) to load EXT This drug carrier material presents several beneficial properties for sustained-release drug delivery, including controllable size and pore morphology to obtain the desired rate of drug release, large internal surface area to allow the adsorption and delivery of high drug payloads within the nanopores, proper in vivo stability preventing premature drug degradation, biodegradability and biocompatibility [11–14]. Moreover, SBA-15 has the potential for the delivery of peptides and proteins. For example, cytochrome c, xylanase, and heme proteins have been loaded into SBA-15 [15–17]. Favorably, peptides and proteins loading into SBA-15 avoid stressful procedures that can protect the molecules from bioinactivation, and the stable inorganic oxide framework of mesoporous silica nanoparticles shelters the peptides and proteins from chemical and thermal exposure and harmful species; thus, the electrochemical activity of proteins and peptides would be retained or even increased. Additionally, studies on EXT loaded into SBA-15 are few.

In this study, we have developed EXT loaded into SBA-15 (EXT-SBA-15) delivery system to control the sustainability of EXT. EXT-SBA-15 showed a high drug-loading efficiency. Morphology and pore size of SBA-15 was characterized. The obtained results indicated that EXT-SBA-15 has a significant sustained-release effect and can improve EXT’s pharmacokinetic features. Furthermore, EXT-SBA-15 exhibited long-lasting and remarkable glucose-lowering effects. The present study demonstrates the therapeutic potential of EXT-SBA-15 for clinical applications.

## Methods

### Materials and animals

Exenatide (mw. 4186.63, 98%) was provided by GL Biochem Ltd. (Shanghai, China). Poly(ethylene oxide)–poly(propylene oxide)–poly(ethylene oxide) (PEO_20_-PPO_70_-PEO_20_) (P-123), Tetraethyl orthosilicate (TEOS) and streptozotocin (STZ) were purchased from Sigma-Aldrich Co., Ltd. (St. Louis, MO, USA). Exendin-4 ELISA kit (EK-070-94) was purchased from Phoenix Pharmaceuticals Inc. (USA). Sprague-Dawley (SD) rats (250 ± 20 g) and ICR mice (25 ± 5 g) were supplied by SLRC Laboratory Animal Ltd. (Shanghai, China). All experimental protocols and animal handling procedures were performed in accordance with the guidelines for the care and use of laboratory animals and approved by the Committees for Animal Experiments at Zhejiang University.

### Preparation of EXT-SBA-15

SBA-15 was prepared by hydrothermal method according to the existing methods [18]. SBA-15 used in this study was synthesized according to the following procedure: We mixed 4 g P-123 in 129.6 g double distilled water and 19.3 mL of hydrochloric acid (HCl 37%). Stirring the mixture intensively at 50 °C for 2 h was needed to ensure a homogeneous emulsion. Then TEOS (8.65 g) was added and the system was stirred for another 20 h at 80 °C. The resultant particles were collected by centrifugation (Optima MAX Ultracentrifuge, Beckman-Coulter Co. Ltd., California, USA) (5590×g, 30 min) after washing with 1000 mL of distilled water. The precipitate was dried in a vacuum at 100 °C for 12 h, and then SBA-15 was obtained by calcination at 550 °C for 6 h.

Dispersing 20 mg SBA-15 with 10 mL aqueous solution of EXT (1 mg/mL) and stir at a rate of 300 r/min at room temperature for 24 h. The loading solution was treated with ultrasound 3 times to guarantee homogeneity [19]. The nanoparticles were collected by centrifugation (5590×g, 30 min), followed by washing with double distilled water for three times. Then, EXT-SBA-15 was dried for 3 h at room temperature in vacuum.

EXT loading degree was detected by thermogravimetric (TG) analysis (20 °C/min, 25 °C–800 °C N_2_ gas purge 200 mL/min, TGA, SDT Q600, TA instrument). Three batches of EXT-SBA-15 were used in the experiments.

### In vitro characterization

Morphological evaluation of blank SBA-15 was performed on transmission electron microscopy (TEM, H-7650, Jeol, Tokyo, Japan). Samples were prepared by dispersing the powder products as slurry in water, which was then deposited and dried on a holey carbon film on a copper grid. A low-exposure technique was used to reduce the effect of beam damage and sample drift. The micrographs of SBA-15 were recorded digitally with TEM operating at 200kv. The particle size (mean diameter, nm) and Zeta potential (mV) were determined by Zetasizer Nano-ZS (Malvern Instruments, Malvern, UK) at room temperature.

The mesostructure ordering was recorded on a small angle X-ray diffractometer (SAXRD) analyzer (D8-ADVANCE, Bruker, Karlsruhe, Germany) with the scattering angle (2θ) range from 0.5° to 6° and scanning speed of 0.02°/min.

Surface area, pore size and pore volume were calculated by N_2_ adsorption-desorption isotherms and structure parameters were determined with multi-channel automatic specific surface area analyzer (TriStar II 3020, Micromeritics Instrument Corp, USA). The surface area and pore size of MSNs were calculated by the BET and the Barrett Joyner Halenda (BJH) methods respectively. The pore volume was determined from the absorption branch of the N_2_ isotherm curve at the *P/P*_*0*_ = 0.983 signal point, STP, standard temperature and pressure.

### In vitro release study

5 mg EXT-SBA-15 nanoparticles were placed in an Eppendorf tube and suspended in 1.5 mL phosphate-buffered saline (PBS, pH 7.4). Eppendorf tubes were placed in a water bath with orbital shaking at a frequency of 150 shakes/min at 37 °C. The release medium was removed at each time point (0.04, 0.08, 0.17, 0.25, 0.42, 0.67, 1, 2, 3, 5, 7, 10, 14 d) after centrifugation at 5590×g for 15 min. Supernatants were collected for the High Performance Liquid Chromatograph (HPLC) analysis of the EXT concentration. The system was consisted of an Agilent 1200 (Agilent, USA) equipped with UV detector. The analytical column was a TSKgel G2000SWXL column (300 mm × 7.8 mm, 5 μm) maintained at 25 °C. The mobile phase consisted of 0.13 mol/L sodium sulfate (Na_2_SO_4_) and acetonitrile (75:25) containing 0.1% trifluoroacetic acid (TFA) at a flow rate of 0.8 mL/min, and EXT was detected at 283 nm.

### Pharmacokinetics study

Pharmacokinetic studies of EXT-Sol and EXT-SBA-15 were conducted in healthy male SD rats. Sixteen rats were randomly divided into two groups (*n* = 8) and fasted overnight with free access to water before administration. SD rats were received s.c. administration of EXT-Sol and EXT-SBA-15 at a single EXT dose of 50 μg/kg, respectively [20, 21]. The blood samples (0.5 mL) of EXT-Sol treatment group were collected from the rat’s orbit and at specific time 0, 0.25, 0.5, 1, 1.5, 2, 3, 4, 6, 8 h after injection [21]. The blood samples (0.5 mL) of EXT-SBA-15 treatment group (0.5 mL) were collected at 0, 0.5, 1, 1.5, 2, 3, 5, 8, 12, 24, 36, 48, 72, 120 h. The collected blood samples were centrifuged at 503×g for 10 min to obtain the plasma, which were stored at −80 °C for further analysis. The plasma concentrations of EXT were measured by Exendin-4 ELISA kit according to the instructions supplied by the manufacturer [22–24]. The relevant pharmacokinetic parameters were analyzed by PKSolver 2.0 [25].

### In vivo Pharmacodynamics study

Type 2 diabetes model was induced by STZ with multiple injection method into ICR mice. Each mouse was injected intraperitoneally with 30 mg/kg⋅d STZ in 0.1 mol/L citric acid buffer (pH 5.0) [26, 27]. One week after the injection, mice were fasted for 12 h (with free access to water) before their blood glucose was measured by glucometer (Accu-Chek Performa, Germany). Diabetic mice were considered by fasting blood glucose above 11.1 mmol/L. Six healthy normal ICR mice were used as the blank control group (A). Twenty-four mice that had developed type 2 diabetes were randomly divided into four experimental groups and received s.c. administration of saline (B), EXT-Sol (C), EXT-SBA-15 (D) and SBA-15 (E), respectively. Group A and B received saline twice daily via s.c. administration. Group C received EXT-Sol twice daily with a dose of EXT (3 μg/kg/d). Group D and E were administrated a single injection of EXT-SBA-15 (600 μg/kg) and SBA-15 via s.c. administration, respectively [28]. The blood sample (about 0.2 ml) was collected from the tail-vein on days 0, 1, 5, 10, 15, 20, 25 for all groups, and blood glucose levels were determined measured simultaneously with a glucometer before administration.

### Statistical analysis

Statistical analysis using one way analysis of variance (ANOVA) with SPSS software (version 19, IBM Inc., Chicago, IL, USA) and a value of *p* < 0.05 was considered to be statistically significant.

## Results

### In vitro characterization

**Fig. 1:**
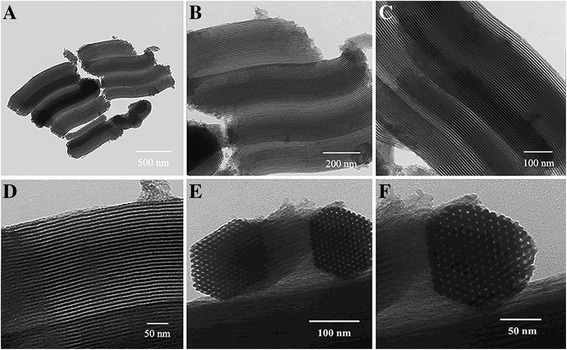
TEM images of SBA-15 with the long axis direction (**a, b, c, d**) and in the direction perpendicular to the long axis (**e, f**)

**Fig. 2:**
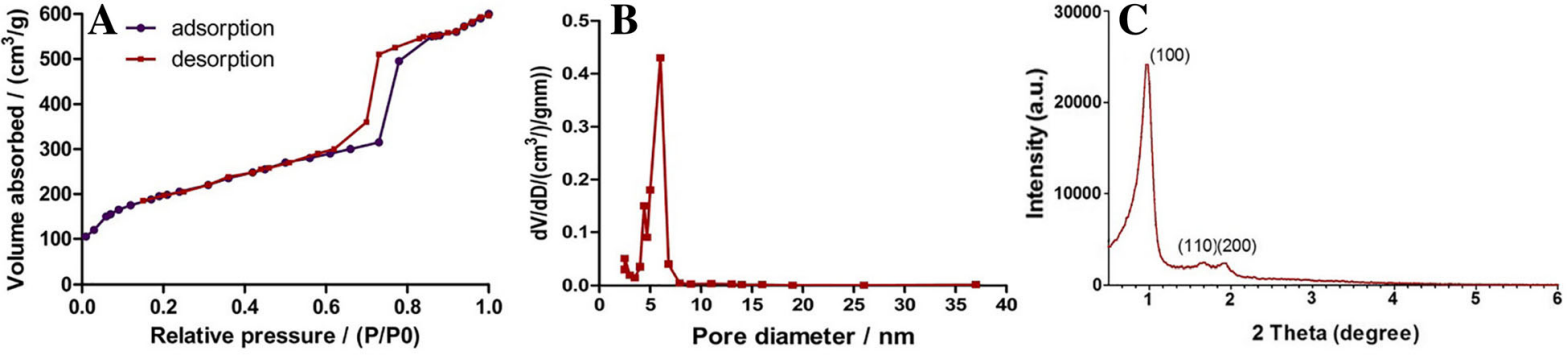
**a** Nitrogen adsorption/desorption isotherm, **b** BJH pore size distribution, **c** and SXRD pattern of SBA-15

Figure 1 a, b, c, d shows that SBA-15 has a rod-like morphology with uniform size and less aggregation. The well-ordered hexagonal arrays of mesoporous on SBA-15 are shown in the highly magnified TEM image (Fig. 1e, f) and confirm that SBA-15 has a 2D p6mm hexagonal structure. The prepared SBA-15 exhibited an average diameter of 920 ± 120 nm with a narrow size distribution, and the Zeta potential is −8.19 ± 2.96 mV. Figure 2a displays the N_2_ adsorption–desorption patterns of SBA-15 exhibiting the characteristic type IV isotherm, and a clear type-H1 hysteresis loop was observed; this observation further proves that mesoporous were regular and uniform. In addition, SBA-15 was yielded with a Barrett Joyner Halenda pore volume of 0.94 ± 0.03 cm^3^/g and a Brunauer Emmett Teller surface area of 701.4 ± 0.70 m^2^/g. The pore size distribution of SBA-15 shows a narrow distribution with a mean value of 6.0 ± 0.2 nm (Fig. 2b). The SAXRD of SBA-15 is shown in Fig. 2c; a strong diffraction peak occurred around 1°, indicating that the internal structures of nanoparticles were in ordered arrangement. On the contrary, the diffraction peak became weak in 1–2°. Three well-resolved peaks are indexable as (100), (110), and (200) reflections associated with a P6mm hexagonal symmetry. TGA method was applied to calculate the drug-loading efficiency (15.2 ± 2.0%).

### In vitro release study

**Fig. 3:**
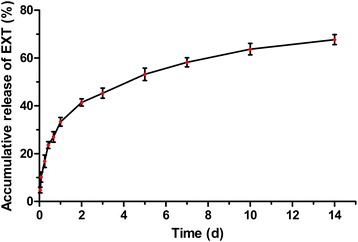
In vitro release profiles of EXT from EXT-SBA-15 during 14 d. (PBS, pH 7.4) (*n* = 3)

The in vitro release profiles of EXT-SBA-15 in 2 weeks were illustrated in Fig. 3. The release rate was relatively rapid, with approximately 33% of the encapsulated drug released at day 1. The rapid initial release of the cargo can be explained by wake bonding between the drug and the surface of the nanoparticles through electrostatic absorption and Van der Waals Forces. Then the drug released more slowly and moderately than before. The cumulative release is about 67% in 14 days. The in vitro release study of EXT-SBA-15 enhanced its sustained-release characteristics and can control the concentration within a narrow range.

### Pharmacokinetics study

**Fig. 4:**
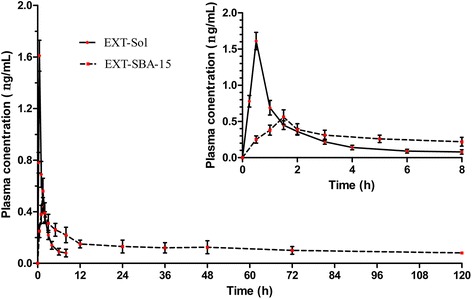
Plasma concentration–time profiles of EXT-Sol (solid line) and EXT-SBA-15(dotted line) after s.c. injection (*n* = 8)

The pharmacokinetics of EXT-SBA-15 was evaluated in SD rats through s.c. administration to investigate the in vivo behavior of EXT-SBA-15. The plasma concentration–time profiles of rats are shown in Fig. 4. After SD rats were injected with EXT-Sol at a dose of 50 μg/kg, the plasma concentration of EXT increased rapidly, reached the peak within 0.5 h, followed by a rapid decrease to baseline after 8 h. In contrast, in the group treated with EXT-SBA-15, the absorption and elimination of EXT in vivo were very slow. Sustained-release characteristics can be observed in EXT-SBA-15. The EXT concentration–time curves for EXT-Sol and EXT-SBA-15 were all fitted with the two-compartment model; the pharmacokinetic parameters are listed in Table 1. The plasma concentration of the sustained-release EXT-SBA-15 attained *C*_max_ of 0.39 ng/mL at *T*_max_ of 1.38 h. The EXT-SBA-15 treatment group extended the half-life *t*_1/2(β)_ to 14.53 ± 0.70 h compared with that of the EXT solution (EXT-Sol) treatment group (0.60 ± 0.08 h) in vivo. Remarkably, a prolonged MRT (*p* < 0.01) indicated the extended circulation time of EXT-SBA-15; the AUC0_**→∞**_ of EXT-SBA-15 was significantly larger than that of EXT-Sol; these results implicated that EXT-SBA-15 possess an enhanced sustained-release characteristics and improves the pharmacokinetic behavior of EXT in rats by prolonging the retention time in circulation.

**Table 1:** Pharmacokinetics parameters of EXT-Sol and EXT-SBA-15 after s.c. injection in SD rats (Mean ± SD, *n* = 8)

Parameters	EXT-Sol	EXT-SBA-15
*t*_*1/2(α)*_/h	0.22 ± 0.03	0.26 ± 0.05*
*t*_*1/2(β)*_/h	0.60 ± 0.08	14.53 ± 0.70**
*t*_max_/h	0.46 ± 0.02	1.38 ± 0.22*
*c*_max_/ng⋅ml^−1^	1.16 ± 0.09	0.39 ± 0.01*
AUC_0→t_/ng⋅ml^−1^⋅h	1.71 ± 0.07	8.60 ± 0.57**
AUC_0→∞_/ng⋅ml^−1^⋅h	1.71 ± 0.07	8.77 ± 0.76**
MRT/h	1.14 ± 0.07	21.30 ± 0.99**

*p < 0.05, **p < 0.01, EXT-SBA-15 compared to EXT-Sol

*t*_*1/2(α)*_ half-life of distribution, *t*_*1/2(β)*_ half-life of elimination, *t*_max_ time to peak concentration, *c*_max_ peak concentration, *AUC*_*0→t*_ area under concentration-time curve from 0 to final time, *AUC*_*0→∞*_ area under concentration-time curve from 0 to infinite time, *MRT* mean residence time of drug in body

### In vivo Pharmacodynamics study

**Fig. 5:**
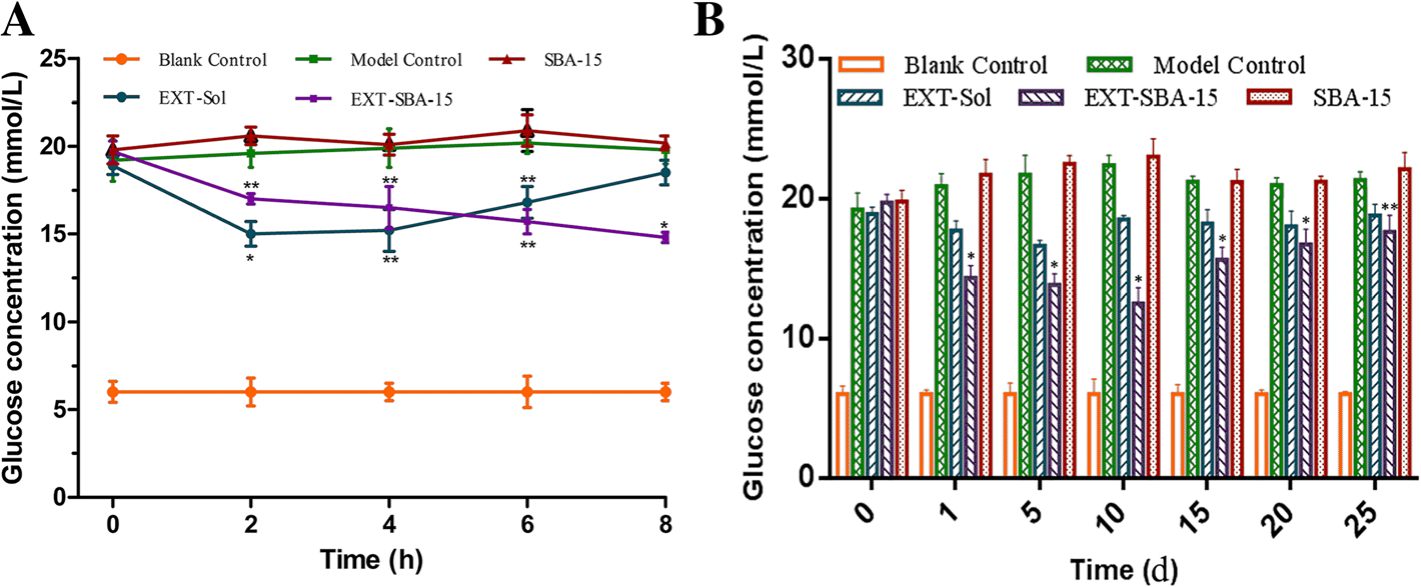
The anti-diabetes effects of EXT-Sol and EXT-SBA-15 on diabetic mice (*n* = 6). **a** The short-term glucose regulating effects of EXT-Sol and EXT-SBA-15 on diabetic mice (s.c. EXT 3 μg/kg/d, twice daily; EXT-SBA-15600 μg/kg, single injection, *n* = 6). **b** The long-term glucose regulating effects of EXT-Sol and EXT-SBA-15 on diabetic mice (s.c. EXT 3 μg/kg/d, twice daily; EXT-SBA-15600 μg/kg, single injection, *n* = 6). *P < 0.001 and **P < 0.01 compared to model control

As shown in Fig. 5a, during the first 8 h after treatment, the blood glucose levels of saline-treated diabetic mice (model control treatment group) were at 20 mmol/L, whereas the group treated with EXT-Sol effectively lowered blood glucose within 2 h and EXT-Sol was kept effective for another 2 h, and then blood glucose gradually returned to the original level. By contrast, the group treated with EXT-SBA-15 slowly reduced the blood glucose levels to 14 mmol/L, which exhibited long-lasting and remarkable glucose-lowering effects. These results indicated a prolonged duration of action in the case of EXT-SBA-15 treatment. In addition, the blood glucose concentration in the blank SBA-15 treatment group was kept greater than 20 mmol/L after s.c. treatment; this result indicates that blank SBA-15 had no effect in lowering glucose. During further measurements, up to 25 days (Fig. 5b), the effect on glucose lowering was not observed in the EXT-Sol treatment group in spite of twice daily injection. However, the EXT-SBA-15 treatment group continued to inhibit glucose level after administration and had a significant difference with the model control treatment group (*p < 0.001, **p < 0.01, compared with model control treatment group). The lowest blood glucose level was 13 mmol/L on the 10th day. Similarly, the SBA-15 treatment group did not have an effect of lowered glucose level.

## Discussion

EXT is a very promising drug for the treatment of type 2 diabetes. However, due to the rapid degradation of EXT in vivo via elimination in the kidneys, it must be administered continuously twice-daily s.c. injection. The need for EXT has continued to fuel attempts to develop it with long-acting pharmacodynamic and pharmacokinetic properties. Therefore, SBA-15 might offer a solution to the obstacles of EXT administration. In the present study, the large-pore SBA-15 had high payloading (15% *w*/*w*), though EXT is a large-molecule drug. Nanoporous structure and large surface area of SBA-15 contributes to its great capability to adsorb and deliver high amounts of EXT that has been a problem with traditional polymer materials (3.65% *w*/*w*) [29]. Furthermore EXT consists of a 39-amino acid, which might be loaded into nanoporous SBA-15 in the form of a chain structure. Hence, the rod-like SBA-15 is a potential to load EXT. EXT adsorption into nanoporous SBA-15 is caused by a complex interplay of different mechanisms, such as the electrostatic interaction, counterion release, and Van der Waals Forces [30]. The protein molecules bind to the negative-charged silica via charged amino acid residues on the protein’s surface. In addition, as the concentration of EXT outside was much higher than that inside, EXT tended to diffuse into the channel of the nanoparticles. Therefore, only by combining different mechanisms are we able to explain the load behavior of EXT into SBA-15.

The in vitro drug release of EXT-SBA-15 exhibited biphasic drug release patterns, which is a burst release at the initial stage and a prolonged release afterwards. The initial burst release of EXT, which benefits by quickly reaching the effective treatment concentration, was due to the presence of EXT in the external pores of SBA-15. EXT in the pores inside SBA-15 were released slowly into the release medium and maintained the effective concentration. As mentioned earlier, the release of drug stored in mesoporous silica nanoparticles occurs only after the release medium has penetrated into the channels and the drug has been dissolved. This is then followed by diffusion along aqueous pathways into the medium. The mechanism of release is a combination of diffusion and dissolution processes [31, 32]. EXT molecules, which are loaded in large-pore SBA-15, had much more opportunity of escaping from pore channels and diffusing into the release medium. Furthermore, the release rate of EXT from SBA-15 may depend on the degradation of the silica nanoparticles. Silica nanoparticles degrade over time in the body, which is available to be material for drug delivery [33]. The in vitro release and pharmacokinetics studies demonstrate sustained EXT release from the nanoporous SBA-15. Pharmacokinetics results suggest that EXT-SBA-15 significantly improved the absolute bioavailability of EXT from that of the EXT-Sol. These results suggest that a complete absorption of subcutaneous EXT requires a sustained release process instead of an immediate release process. Combined with the in vitro drug release study, the cumulative release rate (*F*_*r*_) was the independent variable, and the in vivo absorption percentage (*F*_*a*_) was the dependent variable. The correlation equation was *F*_a_ = 1.152*F*_r_ − 3.137, *r* = 0.8161. EXT-SBA-15 showed certain correlation between in vitro cumulative release rate and in vivo absorption percentage. Generally, the pharmacokinetics results indicate that the investigated SBA-15 nanocarriers are compatible with the peptide and do not compromise its biological activity, as the method used here for determining EXT plasma concentrations is a specific ELISA-detecting active EXT molecules.

The pharmacological activity of EXT, which was administered via SBA-15 nanoparticles in vivo, was examined by measuring the glucagon level. In agreement, sustained effects on glucagon inhibition were obtained with EXT-SBA-15 nanoparticles. The short-term glucose-regulating effects of EXT-Sol and EXT-SBA-15 on diabetic mice confirmed a more rapid and immediate decrease in the blood glucose after EXT-Sol administration compared with EXT-SBA-15 administration. However, the effect of EXT gradually weakened after 2 h. EXT-SBA-15 exhibited efficacy that is comparable to EXT-Sol on 5 h. Finally, EXT-Sol returned to the original glucagon level after 10 h; this result shows that EXT has been metabolized completely in vivo and has no ability to control blood glucose for a long time. The long-term glucose-regulating effects of EXT-SBA-15 administration showed that on the first day, a relatively rapid decrease in the blood glucose level was observed. This result indicates that the initial release rate of EXT-SBA-15 is adequate for its pharmacological efficacy. In the first week after EXT-SBA-15 administration, glucose level is maintained at a lower concentration level, and the lowest blood glucose level was 13 mmol/L on the 10th day. Afterwards, the blood glucose concentration started to increase; however, the glucose level was significantly lower than that of the model control group (>20 mmol/L). These results clearly indicate that EXT-SBA-15 provides adequate initial release and sustained release in vivo to maintain blood glucose levels.

## Conclusion

In conclusion, EXT was successfully loaded into SBA-15 mesoporous silica nanoparticles with a high loading degree, as much as 15% *w*/w. In vitro release and pharmacokinetics studies EXT showed sustained release from the nanoporous of SBA-15. It exhibited prolonged circulation time and significantly increased the bioavailability of EXT. In addition, EXT-SBA-15 showed a prolonged hypoglycemic effect compared with EXT solution. These works contribute to improve EXT clinical use, and further develop the SBA-15 as drug carriers for achieving controlled proteins and peptides drug delivery.
